# Inhibition of casein kinase 1δ/εimproves cognitive-affective behavior and reduces amyloid load in the APP-PS1 mouse model of Alzheimer’s disease

**DOI:** 10.1038/s41598-019-50197-x

**Published:** 2019-09-24

**Authors:** S. Sundaram, S. Nagaraj, H. Mahoney, A. Portugues, W. Li, K. Millsaps, J. Faulkner, A. Yunus, C. Burns, C. Bloom, M. Said, L. Pinto, S. Azam, M. Flores, A. Henriksen, J. Gamsby, D. Gulick

**Affiliations:** Department of Molecular Medicine and Byrd Institute, University of South Florida, Tampa, FL USA

**Keywords:** Alzheimer's disease, Circadian regulation

## Abstract

Circadian rhythm disruption is one of the earliest biomarkers of Alzheimer’s disease (AD), and there exists a bidirectional relationship by which dysfunctions in the circadian clock drive AD pathology and AD pathology drives circadian dysfunction. Casein kinase 1 (CK1) isoforms ε and δ, key circadian regulators, are significantly upregulated in AD and may contribute to AD pathogenesis. In the current studies, we have examined how inhibition of CK1ε/δ with PF-670462 (at 10 mg/kg, δ isoform selective, or 30 mg/kg, δ and ε selective) impacts regional Aβ and circadian gene expression in 10–13 month old APP-PS1 mice and nontransgenic controls. We have also assessed circadian, cognitive, and affective behavioral correlates of these neural changes. At baseline, APP-PS1 mice showed a short period, as well as impaired cognitive performance in both prefrontal cortex and hippocampus-dependent tasks. Both doses of PF-670462 lengthened the period and improved affect, whereas only the higher dose improved cognition. Further, PF-670462 treatment produced a dose-dependent reduction in amyloid burden – overall Aβ signal decreased in all three areas; in the prefrontal cortex and hippocampus, PF-670462 also reduced plaque size. Together, these findings support chronotherapy as a potential tool to improve behavior in AD.

## Introduction

Dementia – a class of neurological disorders characterized by a progressive impairment in cognition and behavior most commonly affecting the elderly – affects nearly 30 million patients and their caregivers, and this number is expected to double over the next 20 years^1^. Treatments for the most common cause of dementia, Alzheimer’s disease (AD), remain elusive, and estimates suggest that as many as 1 in 85 people may suffer from AD by 2050, if no new advances in treatment are made. Patients with AD suffer from irreversible, progressive neurodegeneration and memory loss, and current interventions produce only modest delays in the progression of the disease. Furthermore, few potential treatments for AD make it to clinical trial, and recent drugs that have made it to this stage have failed. One potential explanation for the high failure rate of AD therapeutics is that the intervention occurs too late in the disease – neurodegeneration is rampant by the time clinical symptoms manifest^2^. Thus, the ‘preclinical’ stage of AD represents the optimal treatment time to prevent neuronal loss and progression to the clinical stage of the disease. Therefore, it is essential to identify interventions that may protect neuronal structure and function at this early stage.

Disruption of circadian rhythms and sleep physiology is common in AD, manifesting in symptoms such as altered sleep cycles and body temperature rhythms. Furthermore, the abnormal behaviors associated with AD also show a circadian rhythm, worsening late in the day, and these evening episodes – known as sundown syndrome - are one of the most commonly cited reasons for institutionalization^3,4^. More salient to early interventions in AD, recent studies have demonstrated that circadian rhythm disorders occur years before clinical symptoms, and loss of rhythmicity contributes to the early accumulation of β-amyloid peptide and later cognitive deficits, as well as to neurodegeneration via changes in cellular metabolism, oxidative stress, and autophagy. Interestingly, there is significant crosstalk between the circadian clock and canonical AD pathways. For example, the circadian clock governs rhythms in the activity of both mTORC1 and SIRT1, which have been implicated in the disease. Together, this suggests that circadian rhythm dysfunction may be not only an early biomarker for AD, but also a novel target for interventions to slow the progression of the disease.

The circadian clock is an endogenous timekeeping system designed to control the timing of numerous molecular and physiological processes. This system is highly conserved, allowing organisms from fungi to mammals to anticipate and adapt to daily changes in the environment. At the network level, the master circadian pacemaker, known as the suprachiasmatic nucleus (SCN), resides in the anterior portion of the hypothalamus and functions to maintain the circadian timing of peripheral clocks in the brain and body via neuronal and hormonal output., at the cellular levels, a molecular transcriptional-translational feedback loop (TTFL) control circadian timing in all nucleated cells. At the core of this TTFL, the Brain and Muscle ARNT-Like 1 (BMAL1) and Circadian locomotor output cycles kaput (CLOCK) transcription factors drive the expression of the *Period* (*Per*) and *Cryptochrome* (*Cry*) gene families, which then feedback to repress their own transcription. Additionally, many kinases post-translationally modify core clock proteins to maintain the appropriate timing of the molecular clock. Two such kinases are the serine-threonine casein kinases 1 (CK1) isoforms CK1ε and CK1δ, and each isoform performs a distinct role in the maintaining homeostasis of the molecular circadian clock^5–8^. Specifically, genetic ablation of CK1δ alters the period of the rhythm (the length of a single cycle of sleep-wake), and loss of CK1ε results in arrhythmicity^6,9^. Either situation leads to aberrant behavioral patterns as the clock cycles out of sync with environmental cues. This may be one reason that individuals with AD are phase delayed^10^ - they typically go to sleep later than the rest of the population.

The hallmarks of AD pathology include aggregation of β-amyloid (Aβ) protein into amyloid plaques early in the disease, and hyperphosphorylation and aggregation of tau protein into neurofibrillary tangles in later stages. In addition, both CK1 ε and δ isoforms are upregulated in AD, with CK1δ levels reaching 30x normal^11^, as opposed to a 2.5fold increase in CK1ε^12^. Disruption of the clock by CK1 hyperactivity and changes in PER degradation may be one of the causative factors in the pathological cascade that underlies AD. Work *in vitro* has demonstrated that CK1ε phosphorylates tau, the Aβ precursor amyloid precursor protein (APP), and both β and γ secretases that cleave APP^12,13^, while CK1δ phosphorylates tau^14^. Overexpression of CK1ε increases the secretion of Aβ as may CK1δ, based on the ability of inhibitors selective for the latter to decrease Aβ production^15^. CK1 hyperactivity may also drive a more global circadian disruption and changes in the sleep-wake cycle., Both aging-^16^ and AD-related circadian shifts disrupt slow-wave sleep, which is essential for the clearance of both Aβ and tau^17,18^.

To date, no studies have examined whether inhibition of CK1ε/δ can improve rhythmicity and/or cognition and affect in a mouse model of AD pathology. The current studies test the efficacy of the CK1ε/δ inhibitor PF-670462 to rescue behavior and pathology in the APP-PS1 mouse. PF-670462 readily crosses the blood-brain barrier to restore rhythmicity in mouse models of circadian dysfunction, and has the added benefit of selectively inhibiting the δ isoform at a low dose (10 mg/kg) but inhibiting both δ and ε isoforms at a higher dose (30 mg/kg)^19^. Based on the long-term goal of early identification and rescue of circadian dysfunction in AD, we selected the APP-PS1 Aβ model, as amyloid pathology most often precedes and contributes to tauopathy and memory loss^20,21^. Furthermore, Aβ and tau pathology follow different courses – amyloid first spreads from the prefrontal cortex to the hippocampal formation in the medial temporal lobe, whereas tau spreads from the entorhinal cortex through the rest of the medial temporal lobe then out to more distal cortical regions. Thus, interventions that target tau may be too late to prevent circadian dysfunction and the effects of amyloid pathology in the cortex and hippocampus. We assessed the effects of PF-670462 in three areas. First, we used a circadian locomotor phenotyping system to assess rescue of activity rhythms in a normal light:dark cycle and in constant darkness, which forces the animal to rely on its internal circadian clock. Next, we assessed working and long-term memory, anxiety and affect. Finally, we collected tissue from the prior experiments and examined both PER1 and CK1δ expression as well as amyloid burden in hypothalamic, cortical, and hippocampal regions. Together, our findings support chronotherapy with CK1ε/δ inhibitors to improve behavior, slow disease progression, and enrich quality of life in AD.

## Results

### PF-670462 lengthens the short circadian period defect in the APP-PS1 line and delays activity offset

**Figure 1:**
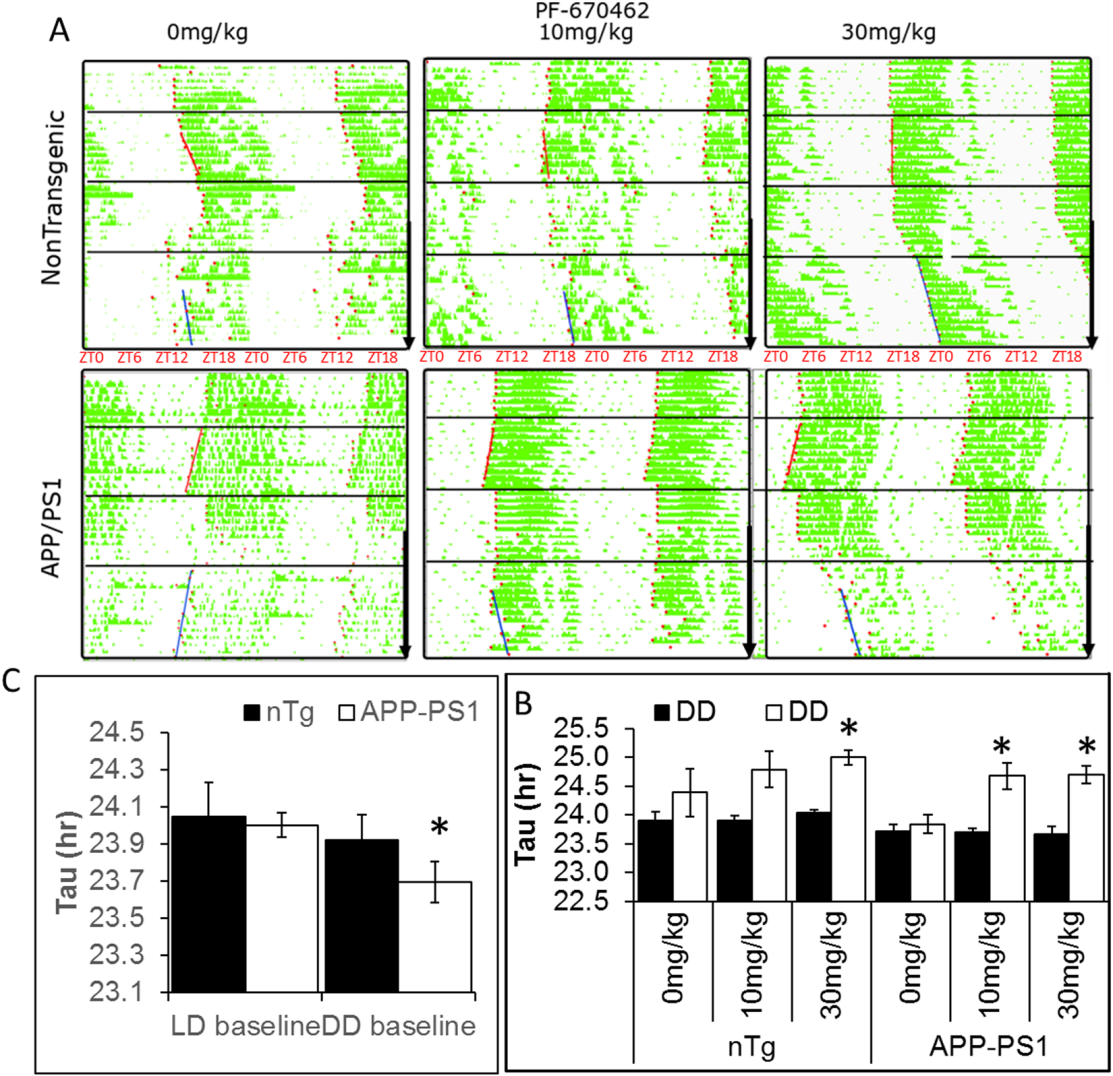
APP-PS1 mice have a short circadian period, and PF-670462 lengthens the period in both APP-PS1 mice and nontransgenic (nTg) controls. (**A**) Representative double-plotted actograms for APP-PS1 and control mice treated with 0, 10, or 30 mg/kg PF-670462. Mice were entrained to a 12:12 light-dark (LD; first segment) cycle for approximately 8 days prior to release into constant darkness (DD; second segment) then returned to LD for 7 days of re-entrainment. On the 8^th^ day of the return to LD, PF-670462 or vehicle treatment at ZT10 was started, and continued for the rest of the experiment (black arrow). Finally, mice were released back into DD for 12 days (fourth segment). ZT values in red indicate the time based on lights-on (ZT0 and lights-off (ZT12) during LD segments of the experiment. (**B**) APP-PS1 mice show a short free-running period in comparison to their nontransgenic littermates in DD. (**C**) PF-670462 lengthened the circadian period in DD (Exptl) compared to the baseline period in each group. Both doses significantly lengthened the period in APP-PS1 mice, while only the higher dose lengthened the period in controls. Data are presented as mean ± SEM. (N = 7–8 per group represented in (**C**) *P < 0.05 compared to controls in (**B)** or compared to baseline mean in (**C**).

We first assessed the baseline rhythmicity in APP-PS1 mice vs. nontransgenic (nTg) controls in 12:12 light dark cycles (LD) and in constant darkness (DD). We found no difference between genotypes in 12:12LD, but found a significantly shorter period in APP-PS1 mice (23.69 vs. 23.95; F(1,49) = 5.21, p < 0.01) in DD (Fig. 1A,B). In LD, there was a significant effect of drug treatment (F(2,48) = 4.10, p < 0.05) and an interaction between experimental phase and drug treatment (F(2,48) = 5.82, p < 0.01) on period, although only 30 mg/kg PF-670462 significantly lengthened the period (data not shown). There were also significant effects of genotype (F(1,49) = 4.21, p < 0.05), drug treatment (F(2,48) = 10.56, p < 0.05) and experimental phase (F(1,49) = 19.07, p < 0.001) and an interaction between experimental phase and drug treatment (F(2,48) = 5.88, p < 0.01) on period in DD. Post-hoc tests demonstrated that APP-PS1 mice maintained shorter periods across treatments compared to nontransgenic controls, and both doses of PF-670462 significantly increased the length of the circadian period in DD, with no significant dose-dependence in this effect in APP-PS1 mice (Fig. 1A,C).

**Figure 2:**
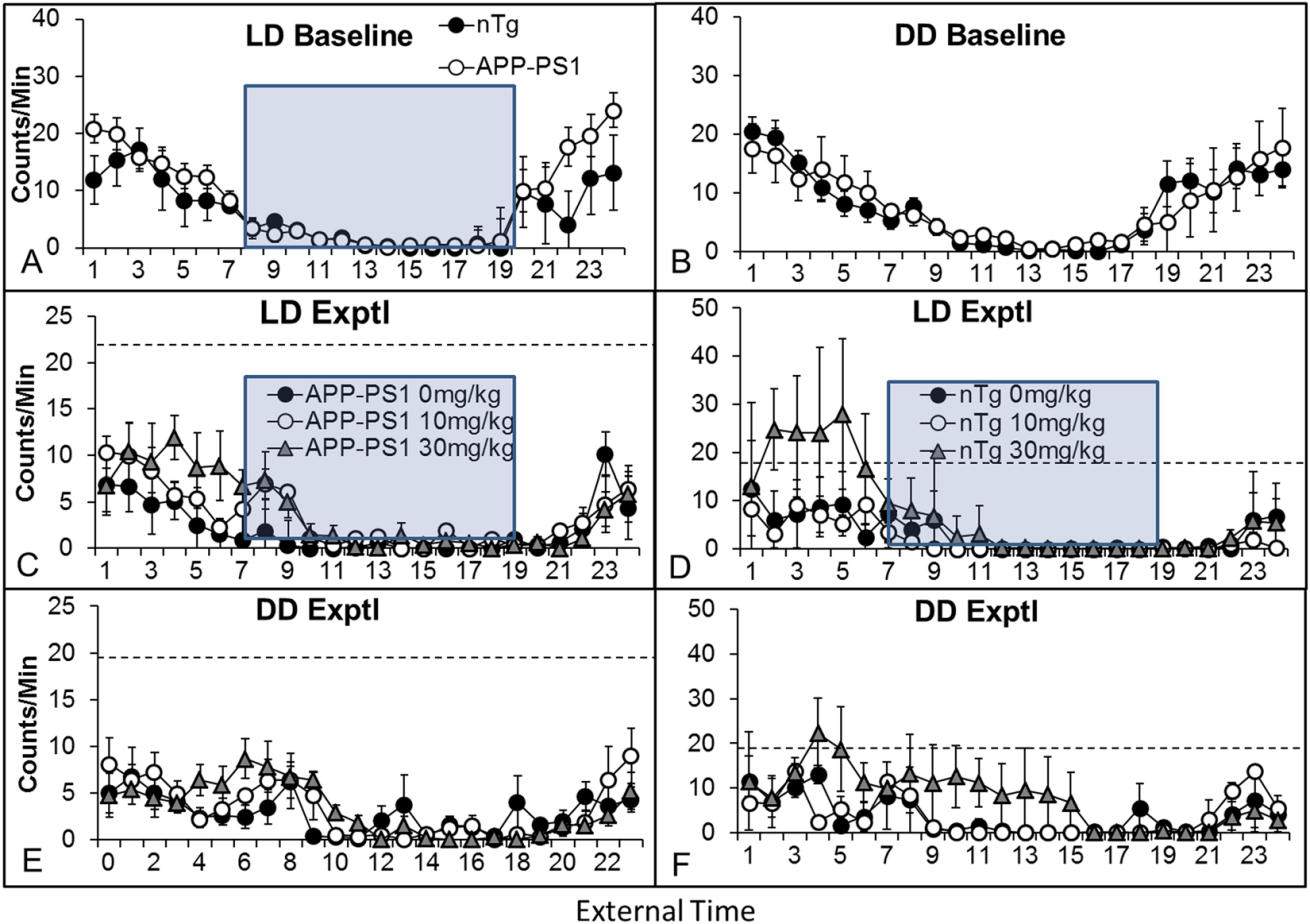
APP-PS1 mice show a more severe collapse of the circadian activity rhythm over time than nontransgenic controls, but PF-670462 fails to rescue the behavioral rhythm. (**A**,**B**) 24 hr activity counts for APP-PS1 and nontransgenic control (nTg) mice in baseline phases of LD and DD. Blue boxes indicate lights-on (sleep phase) in LD. No significant differences were found between groups. (**C**,**E**) APP-PS1 mice show dramatic (61%) drop in activity over the 2 month experimental period in both experimental phases of LD and DD, and this loss in activity in partially reversed (p = 0.06) by PF-670462 in LD. Dotted lines indicate the average counts/min over the 4 hours of peak activity during baseline. (**D**,**E**) Nontransgenic control mice showed a more modest (~35%) drop in activity, and PF-670462 again failed to increase activity despite a trend (p = 0.07) in the group treated with 30 mg/kg PF-670462. Data are presented as mean ± SEM. (N = 7–8 per group).

**Figure 3:**
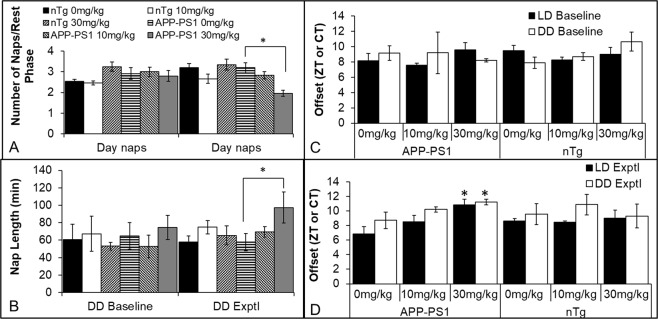
PF-670462 delays sleep onset in APP-PS1 mice. (**A**) APP-PS1 mice treated with 30 mg/kg PF-670462 had fewer naps that lasted longer **(B**) than vehicle-treated controls during the active phase of DD during drug treatment, although total nap time was equal between groups. (**C**) Time of sleep onset (activity below 20% of active phase levels) for APP-PS1 and nontransgenic control (nTg) mice in baseline phases of LD and DD. No significant differences were found between groups prior to drug treatment with all groups ceasing activity approximately one hour after lights-on. (**D**) 30 mg/kg PF-670462 produced an ~3 hr phase delay in sleep onset in APP-PS1 mice in both experimental phases of LD and DD. Data are presented as mean ± SEM. (N = 7–8 per group; * indicates significant difference from the 0 mg/kg group).

We next assessed activity across the experimental phases. Although APP-PS1 mice had greater interdaily variability (more awakenings during the sleep phase and more naps during the active phase) than nontransgenic controls (F(1,49) = 13.80, p < 0.001), there was no effect of experimental phase or treatment group on this measure (data not shown). Thus, we examined activity counts per day. At baseline, there was no difference in overall activity between APP-PS1 and control mice (i.e. no effect of genotype and no interaction of genotype by circadian time), although APP-PS1 mice did show a trend towards a greater amplitude in the activity rhythm in LD, with independent samples t-tests demonstrating greater activity (p < 0.05) at 2200, 2400, and 0100, all times at the peak of the active cycle. There were no differences at any time in DD (Fig. 2A,B). We did find a significant decrease in the activity peak across all groups over time (~61% in both LD and DD in APP-PS1 mice between baseline and experimental phases; 38% in LD and 43% in DD for control mice), but there was only a trend (p = 0.06) towards an effect of drug in LD, with greater activity in mice from both genotypes that were treated with 30 mg/kg PF-670462. We next examined the number and length of bouts of activity during the rest phase and naps during the active phase. There were no group differences during baseline LD or DD, but we did find an effect of PF-670462 on both the number (F(2,48) = 4.60, p < 0.05) and length (F(2,48) = 3.50, p < 0.05) of naps during DD. However, the only difference between groups was that APP-PS1 mice treated with 30 mg/kg PF-670462 had fewer naps that lasted longer than vehicle-treated controls during the active phase of DD, although total nap time was equal between groups (Fig. 3A,B).

Finally, we examined whether PF-670462 might alter sleep timing, based on numerous clinical reports of insomnia and long sleep latency in Alzheimer’s disease. We found a significant interaction of drug treatment by genotype and experimental phase (LD: F(2,48) = 4.70, p < 0.01; DD: F(2,48) = 3.43, p < 0.05). Post-hoc tests demonstrated that 30 mg/kg PF-670462 significantly delayed sleep onset (defined as the first of at least 3 consecutive hours in which wheel counts remained below 20% of the average hourly activity counts in the active phase) in both LD and DD in the APP-PS1 mice compared to vehicle controls, with a 2.5 hr delay in DD and a 4 hr delay in LD (Fig. 3C,D). There were no significant effects in nontransgenic mice or of treatment with 10 mg/kg PF-670462, despite a trend in that direction (p = 0.08) in LD.

Taken together, our data demonstrate that PF-670462 lengthens the short period defect and delays sleep onset in the APP-PS1 mouse, but it fails to consolidate fragmented behavior patterns and also fails to increase the amplitude of the behavioral rhythm.

### PF-670462 reduces exploratory behavior and alters stress-coping behavior

**Figure 4:**
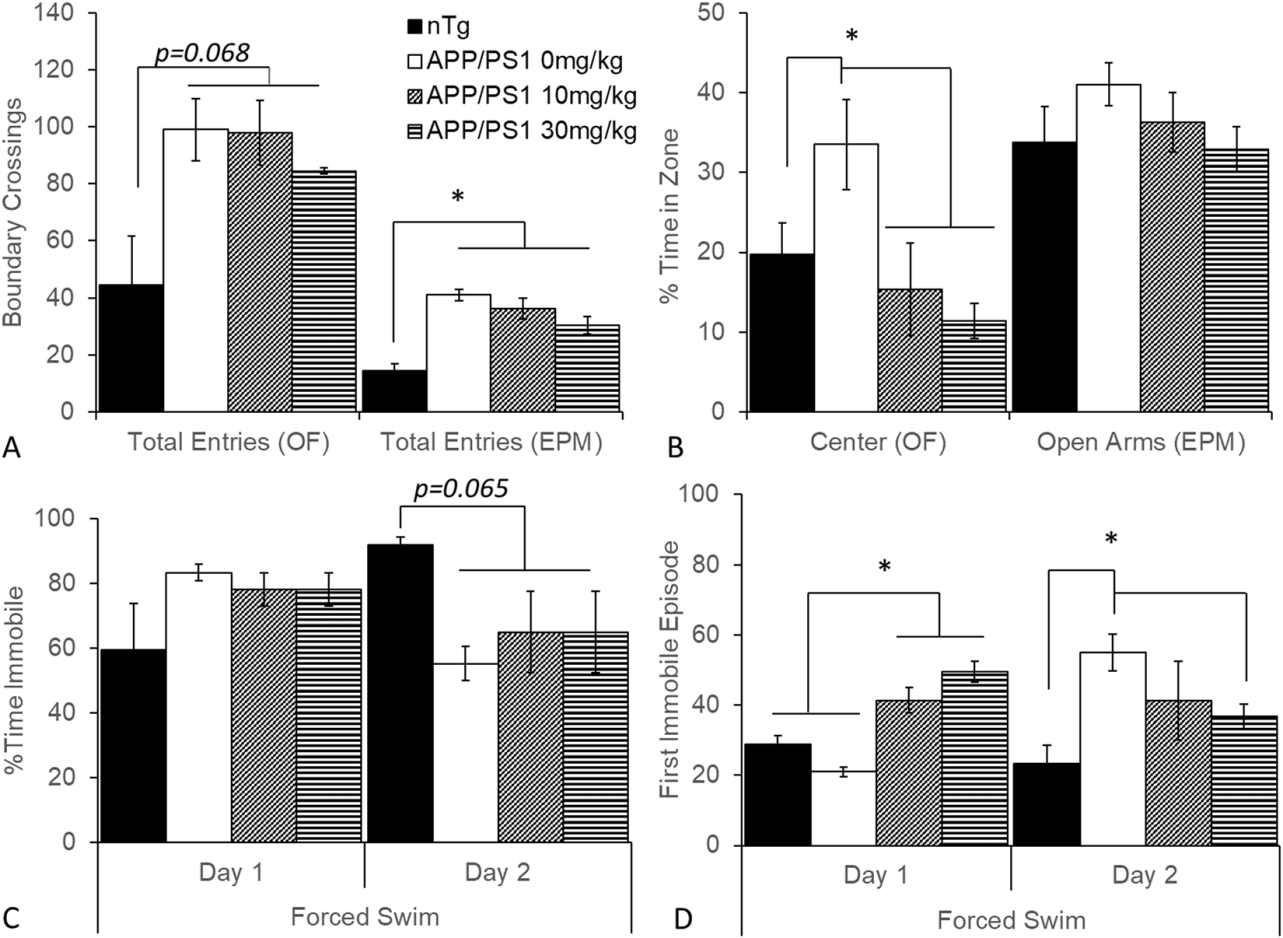
PF-670462 given once daily at ZT10 decreases exploratory behavior and alters depressive behavior in APP-PS1 mice. (**A**) There were no significant effects of PF-670462 on locomotor behavior in the APP-PS1 mice, assessed as the total number of boundary crosses/region zone entries in either open field or elevated plus mazes, although all APP-PS1 were more active than nontransgenic (nTg) mice. (**B**) Vehicle-treated APP-PS1 mice spent more time than nontransgenic controls in the center of the open field, and both 10 and 30 mg/kg PF-670462 reversed this effect, although a similar pattern in time in the open arms of elevated plus maze failed to reach significance. (**C**) There were no differences in the amount of time spent immobile after the first 2 minutes in the forced swim test on either Day 1 or Day 2, although there was a trend towards less time immobile on Day 2 in APP-PS1 mice. (**D**) Both doses of PF-670462 increased the latency to immobility on Day 1 of forced swim compared to both nontransgenic controls and vehicle-treated APP-PS1 mice. Vehicle-treated APP-PS1 mice had greater latency to immobility on Day 2 compared to nontransgenic controls, and 30 mg/kg PF-670462 reversed this effect. Data are presented as mean ± SEM. (N = 4–6 per group; * indicates significant difference).

We first analyzed APP-PS1 behavior in the open field and elevated plus maze tasks, compared to vehicle-treated nontransgenic controls. We found a significant effect of group on the number of boundary crossings in the elevated plus maze (total entries between the zones; F(3,16) = 14.05, p < 0.001) and a trend towards a group effect on boundary crossings in the open field (p = 0.068; Fig. 4A). All APP-PS1 mice had more boundary crossings than nontransgenic mice; despite a trend towards a dose-dependent reduction in crossings with PF-670462, we were unable to detect a significant drug effect. We also found a significant group effect on exploratory behavior. There was a significant effect on the percent time in the center of the open field maze (F(3,16) = 5.73, p < 0.01) but no effect on the percent of time in the open arms of the elevated plus maze, despite a similar pattern between tasks. Vehicle-treated APP-PS1 mice spent significantly more time in the center of the open field maze, and both doses of PF-670462 significantly reduced this behavior (p < 0.05; Fig. 4B). Thus, PF-670462 reduced exploratory behavior without significantly reducing overall activity.

In the Porsolt forced swim test, we found no significant differences in total time immobile in the last 4 minutes of Day 1, although there was a trend towards less time immobile in the last 4 minutes of Day 2 by all APP-PS1 groups (p = 0.065; Fig. 4C). However, we found a significant effect of group on latency to the first immobile episode on both Day 1 (F(3,17) = 3.28, p < 0.05) and Day 2 (F(3,17) = 3.78, p < 0.05). Tukey post-hoc tests revealed that both PF-670462 doses increased the latency to immobility on Day 1 compared to both vehicle-treated nontransgenic and APP-PS1 groups (p < 0.05), whereas vehicle-treated APP-PS1 mice had a longer latency to immobility on Day 2 compared to nontransgenic controls, and 30 mg/kg PF-670462 significantly reversed this latency to immobility (p < 0.05; Fig. 4D). Considering that the forced swim test has recently been reconsidered to model healthy stress-coping strategies, the increased latency on Day 1 and decreased latency suggest an improvement in coping strategies – mice maintain active coping (attempting to find an escape route) longer on Day 1, and switch to passive coping on Day 2 in order to conserve resources in a task they have learned is inescapable.

### PF-670462 improves hippocampus-dependent working and long-term memory, but fails to alter social recognition memory

**Figure 5:**
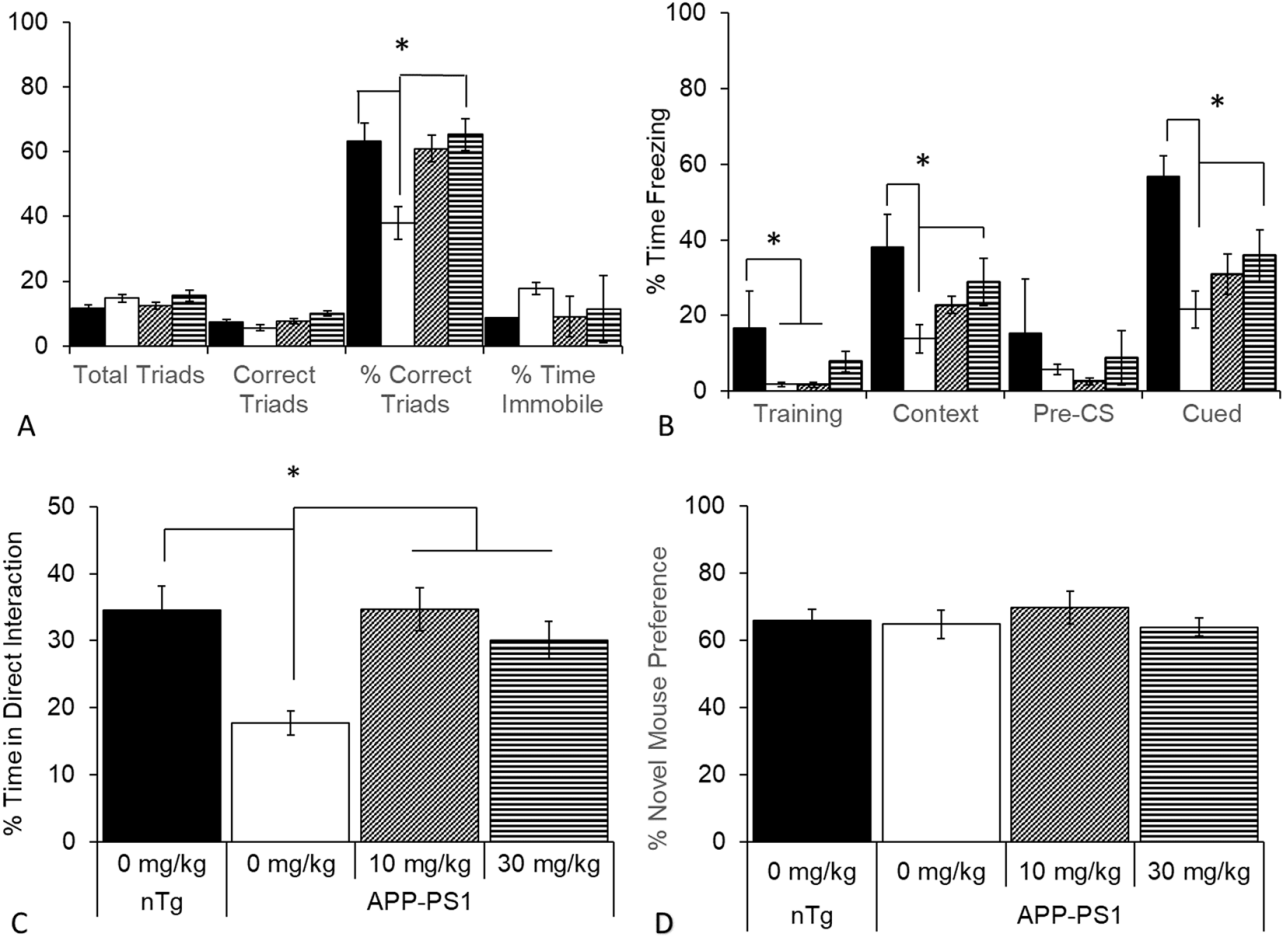
PF-670462 given once daily at ZT10 increases hippocampus-dependent working memory and long-term memory in APP-PS1 mice, but fails to alter social recognition. (**A**) Vehicle-treated APP-PS1 mice achieved fewer correct alternations in the Y-maze spontaneous alternation task, and 30 mg/kg PF-670462 significantly rescued working memory, without altering the overall number of completed triads or the time spent immobile. (**B**) Vehicle-treated APP-PS1 mice froze less than nontransgenic controls (nTg) following the first tone-shock presentation during the training phase of fear conditioning and in both the hippocampus-dependent context test and during tone presentation (Cued) in the CS test. 30 mg/kg PF-670462 rescued behavior in all three phases of the task (**C**) Vehicle-treated APP-PS1 mice spent less time in direct interaction (nose to pencil cup or climbing) with the bait mice in the first phase of the sociability test compared to all other groups. (**D**) However, there were no differences in social memory, assessed by the preference for the novel bait mouse compared to the familiar bait mouse in the final phase of the test. Data are presented as mean ± SEM. (N = 5–6 per group; * indicates significance).

We next analyzed the effects of PF-670462 on cognition in the Y-maze spontaneous alternation task and in contextual and cued fear conditioning. In Y-maze, we found a significant effect of group on working memory, assessed by the percentage of correct triad alternations (visiting all three arms before returning to a previously-visited arm; F(3,19) = 5.34, p < 0.05) but no differences in the total number of arm entries nor in time immobile. Vehicle-treated APP-PS1 mice has a significant deficit in correct alternations, and 30 mg/kg PF-670462 significantly reversed this deficit (p < 0.05). Despite a trend towards a higher percentage of correct alternations in the group treated with 10 mg/kg PF-670462 (p = 0.06 compared to vehicle-treated APP-PS1 mice, no difference from nontransgenic controls), this group was not statistically different from the other two groups (Fig. 5A). In fear conditioning, mice are trained to associate an aversive foot-shock with both a novel chamber and a tone auditory cue. The context-shock association depends on the hippocampus and amygdala, whereas the cue-shock association is hippocampus-independent^22^. We found a significant effect of group (F(3,15) = 5.96, p < 0.01) on freezing following the first tone-shock pairing during training, with both vehicle- and 10 mg/kg-treated APP-PS1 mice freezing less than nontransgenic controls (p < 0.05), and 30 mg/kg PF-670462 partially rescuing this behavior, with no significant differences from any other group. We also found a significant group effect on freezing in the context test (F(3,15) = 6.59, p = 0.01), and during tone presentation in the cue test (F(3,15) = 4.87, p = 0.01). In both tests, vehicle-treated APP-PS1 mice froze significantly less than nontransgenic mice, and 30 mg/kg PF-670462 significantly rescued these deficits (Fig. 5B). Together, we found that 30 mg/kg PF-670462 improved hippocampus-dependent spatial working and long-term memory and hippocampus-independent memory, but the 10 mg/kg dose failed to alter memory, despite an equal rescue of the circadian period by both doses in the circadian locomotor studies.

In the first phase of the 3-chamber sociability test, we found a significant group effect on the percent time spent in direct interaction with the single bait mouse (F(3,18) = 6.69, p < 0.01, Vehicle-treated APP-PS1 mice spent less time in direct interaction than nontransgenic controls, and both doses of PF-670462 increased the time in direct interaction compared to the vehicle-treated APP-PS1 group (p < 0.01; Fig. 5C). There was no effect of PF-670462 on the percent of time spent with the novel mouse compared to the familiar mouse in the second phase of the test, likely due to the absence of any deficit in the vehicle-treated APP-PS1 mouse (Fig. 5D). Thus, while PF-670462 increased social interaction, it did not alter social memory.

### CK1δ is not elevated in APP-PS1 mice

**Figure 6:**
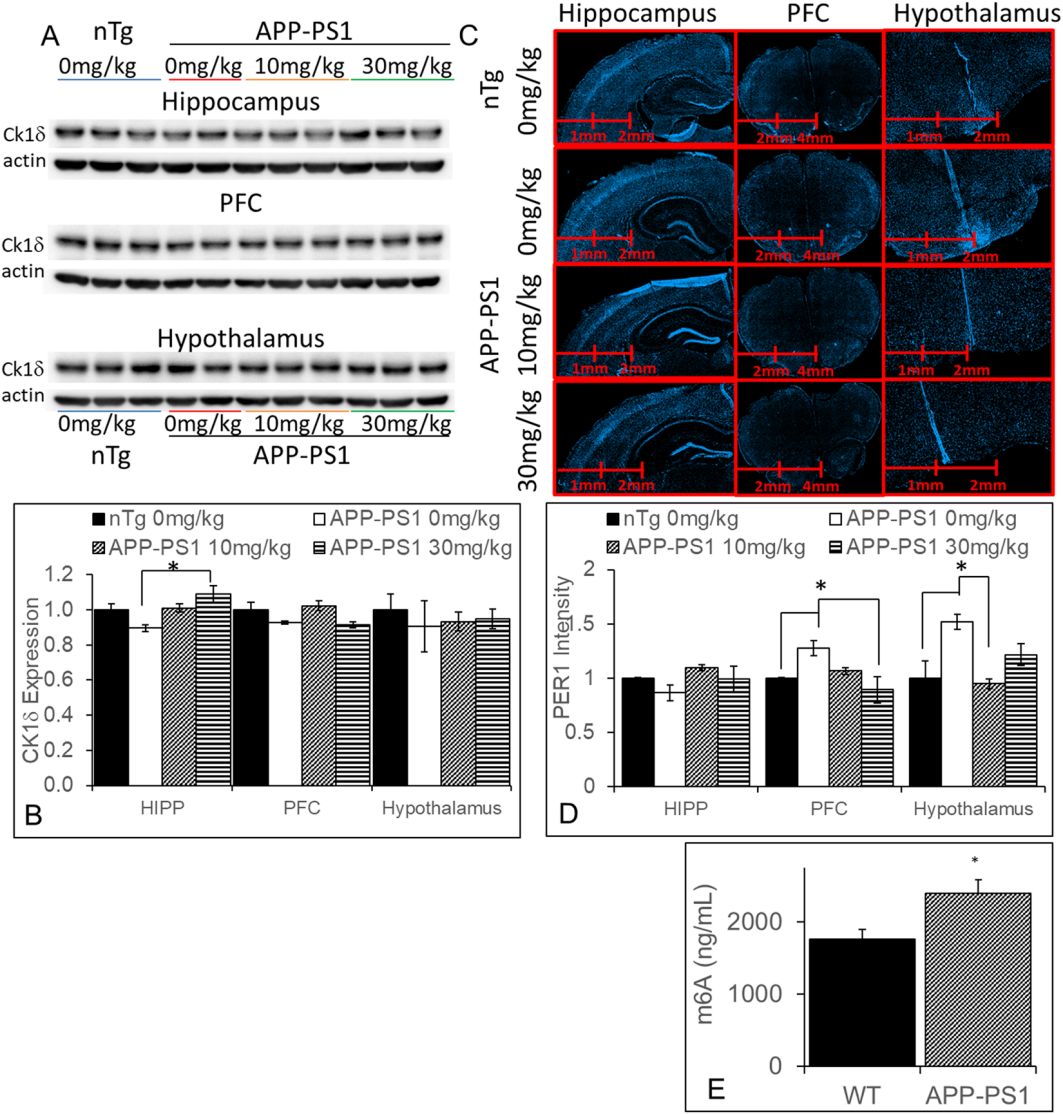
CK1δ is not elevated in the brains of APP-PS1 mice, although CK1 inhibition did alter PER1 expression. (**A**,**B**) Separate Western blots were run for tissue from each brain region. There were no differences in the basal expression of CK1δ between nontransgenic controls (nTg) and vehicle-treated APP-PS1 mice, although 30 mg/kg PF-670462 given once daily at ZT10 did increase the expression of CK1δ in the dorsal hippocampus of APP-PS1 mice compared to vehicle-treated APP-PS1. Samples were cropped between the CK1δ band and the actin bands from the same blot for each brain region. (**C**,**D**) At 200x magnification, there were no differences between groups in PER1 expression at ZT10, although PER1 was increased in the prefrontal cortex and anterior hypothalamus of vehicle-treated APP-PS1 mice compared to nontransgenic controls, and PF-670462 reversed this effect. Data are presented as mean ± SEM. (N = 3–5 per group; * indicates significance).

In order to explore potential molecular mechanisms by which PF-670472 might be impacting behavior in the APP-PS1 mouse, we first assayed CK1δ levels. Considering that CK1δ is elevated up to 30fold in the AD brain^11^, we hypothesized that a CK1 inhibitor might be effective due to similarly elevated levels in the APP-PS1 brain. However, Western blot revealed no such increases at zeitgeber time (ZT)10 (Fig. 6A). Indeed, although there was a group effect on CK1δ expression in the hippocampus (F(3,12) = 3.97, p < 0.05), the only difference between groups was higher CK1δ expression in APP-PS1 mice treated with 30 mg/kg PF-670462 compared to those treated with vehicle (p < 0.05; Fig. 6B), possibly a compensatory response to the CK1 inhibition. Considering that there was no change in CK1δ expression at ZT10, we also examined PER1 staining at the same time point, in the same groups. We found a significant group effect on PER1 expression in the prefrontal cortex (F(3,21) = 5.62, p < 0.05) and anterior hypothalamus (F(3,21 = 8.47,p < 0.01). Specifically, vehicle-treated APP-PS1 mice had significantly higher PER1 expression than nontransgenic controls and PF-670462 reversed this effect (at 30 mg/kg in the prefrontal cortex and at 10 mg/kg in the hypothalamus; Fig. 6C,D). Although PER1 expression changes may indicated a functional change in the APP-PS1 circadian system, the minimal change in CK1δ expression in the APP-PS1 brain suggests that this is not due to the elevation of CK1 seen in the human AD brain.

### PF-670462 reduces amyloid burden in the APP-PS1and nontransgenic mouse brain

**Figure 7:**
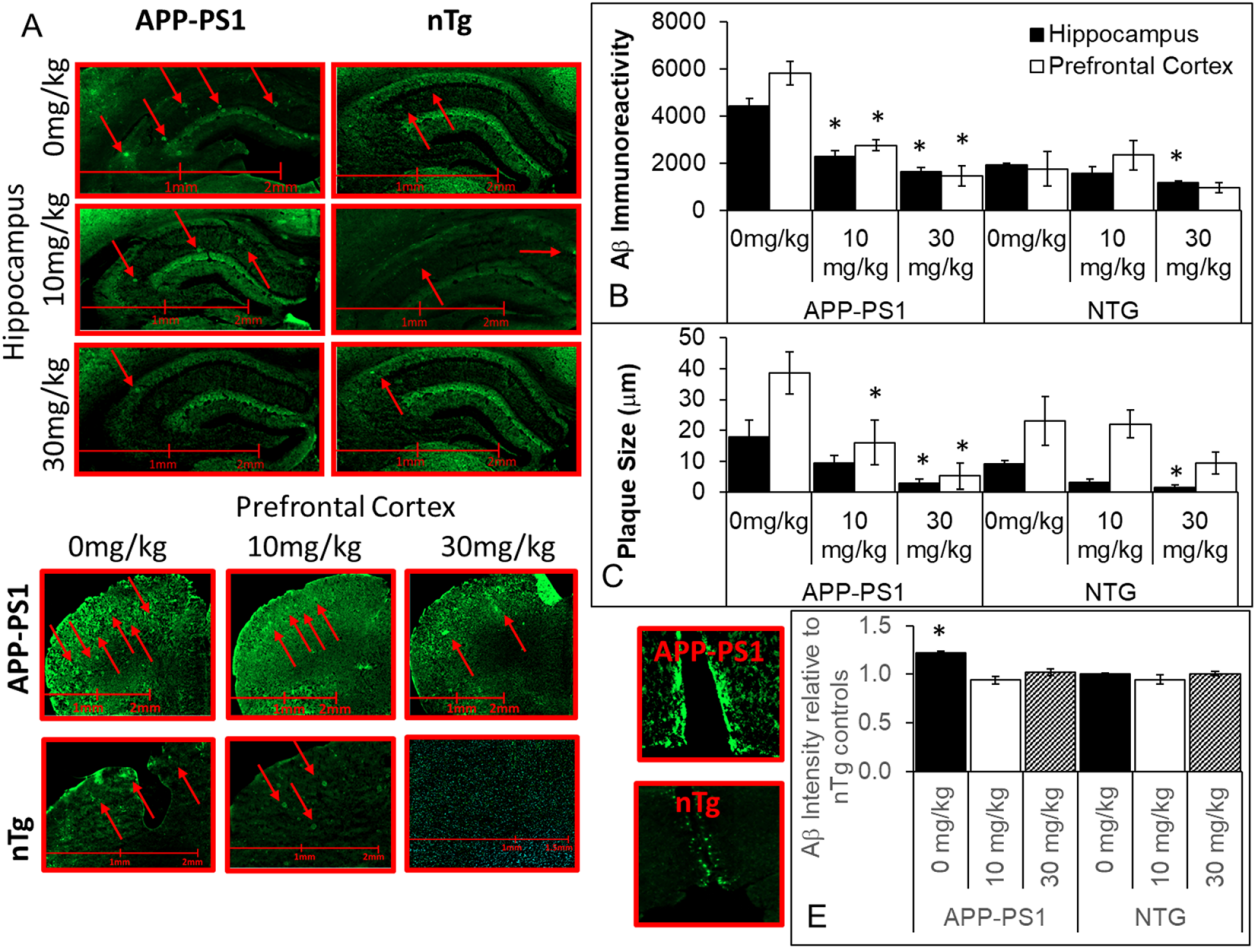
PF-670462 treatment given once daily at ZT10 reduces amyloid burden in APP-PS1 and nontransgenic control (nTg) mice. (**A**) Representative samples of dorsal hippocampal and prefrontal cortical slices at 200x magnification from APP-PS1 and nontransgenic mice treated with vehicle (0 mg/kg), 10 mg/kg, or 30 mg/kg PF-670462. Red arrows indicate plaques. (**B**) Both doses of PF-670462 reduced the overall intensity of staining for Aβ protein across sections sampled from each brain region in the APP-PS1 mice, but the only significant decrease in the aged nontransgenic controls was in the hippocampus following treatment with the 30 mg/kg dose. (**C**) Plaque size (described in μm) was also reduced in both APP-PS1 regions by 30 mg/kg PF-670462, although 10 mg/kg only significantly reduced plaque size in the prefrontal cortex. Again, the only significant decrease in the aged nontransgenic controls was in the hippocampus following treatment with the 30 mg/kg dose. (**D**) Representative samples of the anterior hypothalamus sections containing the SCN from vehicle-treated APP-PS1 and nontransgenic mice. No plaques were identified in the anterior hypothalamus, although some Aβ protein was present. (**E**) Because no plaques were identified in the anterior hypothalamus, overall intensity was normalized to the vehicle-treated nontransgenic group. The only significant difference between groups was higher Aβ staining in the vehicle-treated APP-PS1 group (N = 4–5 per group; *indicates significance from the vehicle-treated group).

The molecular effects of CK1δ and ε are not isolated to the circadian clock. Indeed, CK1ε phosphorylates APP, and both β and γ secretases^12,13^, increasing the secretion of Aβ. Indirect evidence from inhibitor studies also suggests that CK1δ may also decrease the secretion of Aβ^15^. To determine whether PF-670462 may have a direct effect on amyloid burden, we assayed Aβ deposition and plaque size in the hippocampus, prefrontal cortex, and anterior hypothalamus of vehicle and PF-670462 treated nontransgenic and APP-PS1 mice. For Aβ intensity, we found significant effects of brain region (F(2,28) = 7.58, p < 0.001), genotype (F(1,30) = 6.29, p < 0.05), and drug treatment (F(2,28) = 6.70,p < 0.01), and a significant interaction of genotype and drug treatment (F(4, 27) = 4.04, p < 0.05). Tukey post-hoc differences revealed greater intensity of Aβ in the prefrontal cortex and hippocampus than in the hypothalamus (p < 0.05). Furthermore, in the hypothalamus, only the vehicle-treated APP-PS1 mice had higher amyloid load than all other groups (p < 0.05), suggesting that PF-670462 did reduce Aβ in this region in the APP-PS1 brain. We also found greater Aβ intensity in the prefrontal cortex and hippocampus of vehicle-treated APP-PS1 mice compared to controls (p < 0.05), which was significantly reduced by both doses of PF-670462 (p < 0.05). Additionally, the higher dose also reduced intensity in the hippocampus of nontransgenic mice, likely due to the cross-reactivity of the antibody with mouse Aβ (p < 0.05; Fig. 7A,B,D,E). No plaques were identified in the hypothalamus, and so only staining intensity was examined across all three brain regions. We found a significant effect of brain region (F(1,30) = 6.48, p < 0.001) on plaque size, with larger plaques in the prefrontal cortex than hippocampus across groups. We also found significant effects of genotype (F(1,30) = 5.59, p < 0.05), and drug treatment (F(2,28) = 13.60,p < 0.05), and a significant interaction of genotype and drug treatment (F(4, 27) = 3.47, p < 0.05) on Aβ plaque size. Tukey post-hoc differences revealed that vehicle-treated APP-PS1 mice had significantly larger plaques than nontransgenic controls in both regions, and both doses of PF-670462 significantly reduced plaque size in the prefrontal cortex, whereas only the higher dose produced a significant reduction in the hippocampus (p < 0.05). Additionally, the higher dose also reduced plaque size in the hippocampus of nontransgenic mice (p < 0.05; Fig. 7A,C). Together, these data support prior findings of minimal SCN pathology in the AD brain until later Braak stages, and also suggest that PF-670462 reduces amyloid pathology in the APP-PS1 mouse.

## Discussion

Circadian disruptions are common across all stages of Alzheimer’s disease, frequently manifesting in sleep disruptions and sundown syndrome, a constellation of symptoms including confusion, agitation, and wandering in the late afternoon and early evening^23^. In home-care AD patients, caregiver-reported rates of sundowning are as high as 66%^24^. This syndrome reduces quality of life for both patients and caregivers, and is one of the most commonly cited reasons for institutionalization^3,4^. In addition, there are basal circadian rhythms in the molecular mechanisms underlying both cognition^25,26^ and the pathologic changes that disrupt it^27,28^. This suggests that the early disruption of circadian rhythmicity in the AD brain^12^ may play an important role in pathogenesis and in cognitive decline. Thus, the goal of this study was to determine whether pharmacotherapy to reset the circadian rhythm would be sufficient to restore cognitive performance in a mouse model of amyloid pathology.

We found that the lower, δ isoform-selective dose of the small molecular inhibitor PF-670462 was sufficient to lengthen the aberrantly-short free-running period of the aged APP-PS1 mouse, whereas the higher dose, which targets both δ and ε isoforms, lengthened the period in both APP-PS1 and nontransgenic mice. However, the period was not significantly different between doses, in line with prior work demonstrating that the δ isoform determines period. Considering the robust lengthening effect, lower drug doses or a weaker inhibitor will be assayed in future studies. We also found a significantly greater decrease in activity with age in the APP-PS1 mouse compared to nontransgenic controls. Although APP-PS1 mice develop motor deficits, these deficits are mild at the age of testing in the current study^29,30^. Furthermore, APP-PS1 mice displayed normal locomotor ability in the cognitive-behavioral battery. Together, our data and the prior findings suggest that the decreased amplitude in locomotor rhythms is not due to the inability to run on the wheel. Nonetheless, while the wheel-running system is an accurate predictor of circadian rhythmicity, it only measures voluntary activity. As a result, some bouts of activity are missed, and sleep periods can only be inferred from a prolonged absence of activity. Future work should address these shortcomings with telemetry or infrared monitoring. Interestingly, PF-670462 partially reversed the locomotor deficit. Considering that patients with AD are less active than healthy elderly individuals, this improvement in activity may be beneficial clinically. Together, this suggests that drug therapy targeting CK1 may be useful in rescuing AD-related changes in behavior patterns. However, patients with AD generally present with a delayed period (being more active later in the day, and staying up later than normal). Thus, we examined the times of activity onset and offset in these mice. We found no differences in onset, and no genotype differences in basal offset times, but a significant delay on offset time in the APP-PS1 mice treated with PF-670462. Although this also supports the efficacy of changing circadian behavior through CK1 manipulation, the potent lengthening effect of CK1δ inhibition likely masked any beneficial effects of re-entrainment by CK1ε inhibition. Thus, future work will also assay a CK1ε-selective inhibitor, and will use a mouse model which better replicates the AD circadian phenotype, such as the long-period Tg2576 mouse or the nearly arrhythmic 3xTg-AD mouse.

In terms of cognitive and affective behavior, we found that both doses of PF-670462 decreased the aberrantly high exploratory activity of the APP-PS1 mouse without altering total locomotor activity across the elevated plus maze and open field, which might translate to a reduction in wandering behavior in the human scenario. In addition, we found improved coping strategies in the Porsolt forced swim test, which reflects improvements in cognitive flexibility and stress resilience. We also found improved social interaction. Together, these findings support the idea that chronotherapy with PF-670462 could improve affect and social behavior, two common areas for struggle in patients with AD.

Perhaps most promising, the higher dose of PF-670462 also improved spatial working and long-term memory, two prefrontal cortex and hippocampus-dependent measures, and the hippocampus-independent measure of cue-stimulus memory. This suggests a global improvement in cognitive function following treatment with PF-670462, rather than a hippocampus-selective improvement. Indeed, PF-670462 reduced both total Aβ intensity and plaque size in the hippocampus and prefrontal cortex. Despite differing opinions and data in studies examining the correlation between amyloid burden and cognitive decline, many reports demonstrate correlations between the neuropathology and behavioral deficit^31–33^, in agreement with our findings. However, the possibility that PF-670462 is acting via alternative mechanisms cannot be dismissed. Indeed, considering that CK1δ is not elevated in the APP-PS1 brain, there may not be a large role for CK1 in amyloid burden in this model. PF-670462 may instead be improving behavior by normalizing circadian rhythms in cognitive processes, especially considering the drug-mediated changes in PER1 expression in the prefrontal cortex, a brain region necessary for the attention that contributes to all cognitive processes. Previous work has also shown that there is a fully functional and autonomous molecular clock in the hippocampus^34^ that is necessary for hippocampus-dependent learning^35^, and a prefrontal cortical clock that is necessary for cortical contributions to learning^36^. Additionally, the strength of hippocampal long-term potentiation (LTP) shows a circadian rhythm^37^. Together, these findings suggest that simply restoring a basal circadian rhythm may be sufficient to improve learning. Alternatively, reducing even baseline levels of CK1ε/δ may be sufficient to reduce the amyloidogenic effects of this enzyme. In addition, we found an effect of PF-670462 on amyloid burden in nontransgenic controls. Because the antibody that we chose has cross-reactivity in humans and mice, this is likely the result of changes in endogenous amyloidosis in the aging mouse brain.

In summary, PF-670462, a small molecule inhibitor of CK1δ/ε alters rhythmicity, improves affect and cognition, and decreases amyloid load in the APP-PS1 mouse. Together, these findings support the potential efficacy of chronotherapeutic interventions in Alzheimer’s disease. Future work should investigate the prophylactic potential of earlier intervention to reduce CK1ε/δ in mouse models of the disease.

## Methods

### Animals

The studies used male and female APP-PS1 mice and their nTg littermates, 9–10 months old at the beginning of all experiments. Breeding pairs of Tg 2576 APP and PS1 line 5.1 mice were generously provided by Dr. Marcia Gordon, and each genotype was bred in separate lines. APP and PS1 mice were then crossed to avoid phenotypic drift, to produce nTg, APP, PS1, and APP-PS1 mice. Only nTg and APP-PS1 mice were used for these experiments. All breeders and progeny were genotyped as previously described^38,39^. These mice contain human transgenes for both APP bearing the Swedish mutation and PSEN1 containing an M146L mutation, and have progressive amyloid accumulation, beginning in the cortex by 4 months and in the hippocampus by 6–7 months of age^39^. Although these mice develop motor deficits, these deficits are mild or nonexistent at the age of testing in the current study^29,30^. Mice were group-housed with littermates in 12:12 light:dark (LD) cycles unless otherwise indicated. All animal testing procedures and care followed the NIH guidelines and were approved by the University of South Florida’s Institutional Animal Care and Use Committee (Approval ID number A4100–01).

### Drug treatment

Mice were administered vehicle (20% [w/v] 2-hydroxypropyl-β-cyclodextrin buffered with 25 mM sodium citrate pH 6.0; MilliporeSigma, Oakville, ON) or the casein kinase 1δ/ε (CK1δ/ε) inhibitor PF-670462 (10 or 30 mg/kg, Cayman Chemical; Ann Arbor, MI) intraperitoneally (i.p.) once daily at ZT10 (during a 12:12 LD cycle) or CT10 (in constant darkness) based in the prior LD schedule, following the methods of Meng and colleagues^19^.

### Locomotor assessment of circadian activity

Throughout the circadian experiment, male and female mice were individually housed with *ad libitum* access to food and water. Prior to the start of the experiment, mice were maintained on a 12:12 LD cycle. Mice were then moved to running wheel cages (Lafayette Instrument, Lafayette, IN) in light-tight, sound-attenuated cabinets. Activity, measured by revolutions of the running wheel, was recorded in 5 min bins using Scurry Activity Monitoring Software (Lafayette Instrument, Lafayette, IN). To assess free running period in the mice, the lights were turned off at ZT0 (previous lights on time) on day 8 and the mice were subjected to a 24-hour DD for 11 days. The lights were then returned to the 12:12LD cycle for 7 days of re-entrainment. Finally, mice were treated once daily at ZT10 (2 hr before lights off) with PF-670462 (0, 10, 30 mg/kg). Three days into drug treatment, mice were released back into DD for 12 days for the final segment of the experiment. Wheel activity was analyzed using ClockLab Analysis software (ClockLab, Actimetrics, Wilmette, IL) based on our prior work^40^. Activity was quantified as the number of wheel revolutions per minute occurring during 5-min bins. Average activity, interdaily variability (IV) and intradaily stability (IS) were calculated using the batch analysis in ClockLab software. The free-running period (τ) and amplitude were determined by chi-squared (χ^2^) periodogram analysis and onset (waking) times. Offsets (sleep time) were based on the first time in which activity dropped below 20% of average activity for at least 3 consecutive hours; active-phase naps and rest-time activity bouts were calculated as at least two consecutive 30 min bins without activity (nap) or two consecutive 30 min bins with activity (bouts), and the length of each nap or bout was calculated from these data. HOBO Pendant Temperature/Light 8 K Data Loggers (Onset Computer Corporation, Bourne, MA) recorded light and temperature in each cabinet to ensure that no environmental changes occurred outside of the programmed light shifts.

### Cognitive-affective behavioral battery

Throughout the behavioral experiment, male and female APP-PS1 mice were individually housed with *ad libitum* access to food and water and maintained on a 12:12 LD cycle. Mice were treated once daily at ZT10 (2 hr before lights off) with PF-670462 (0, 10, 30 mg/kg). Three days into drug treatment, the behavioral tests began. All behavior was run between ZT2 and ZT6, and mice were brought into the behavior room antechamber 30 minutes before testing began. The order of behavioral tasks was as follows: 1 day of open field, 1 day of 3-chamber sociability, 1 day of elevated plus maze, 1 day of Y-Maze, 3 day break, 2 days of Porsolt forced swim, 1 day break, 2 days of contextual and cued fear conditioning. All mazes were wiped clean with 70% EtOH between trials. For all sessions in which movement between regions was scored, entry into a region occurred when all 4 paws entered that region.

The **open field** task and **elevated plus maze** task were used to evaluate general activity and anxiety levels. Each animal was placed in an open field chamber (27 × 27 cm) for 10 minutes in standard room lighting conditions. All activity in the open field was monitored with an HD camera placed above the apparatus and recorded with the AnyMaze software program (Stoelting, Wood Dale, IL) for hand-scoring. The **elevated plus maze** task consisted of two enclosed arms (30 × 5 × 15 cm) facing each other and two open arms (30 × 5 cm) also facing each other. Each arm is attached to a center platform (5 × 5 cm) and elevated 40 cm off the floor. Two of the runways were well lit and open and the other two were closed, providing protection. Testing was conducted under standard light conditions for 10 minutes, and scored the same way as open field. The **3-chamber sociability task** to assess both social approach vs avoidance and short-term social memory, was conducted within a three-chambered box (60 × 24 cm total) with doors between the chambers. After habituation to the center, a novel (non-littermate, same-sex) bait mouse was placed under a pencil cup in one side chamber, with an empty pencil cup in the opposite chamber. Both doors were then lifted and the subject mouse was free to explore all 3 chambers for 15 minutes. Finally, the subject mouse was returned to the center chamber, the doors were closed, and a second bait mouse was placed under the empty pencil cup. The doors were lifted, and the subject was again allowed to explore freely for 15 minutes. Activity was again recorded and scored. The **Y-maze spontaneous alternation** task to measure spatial working memory apparatus consisted of three arms (38 × 8 cm) at a 120° angle from each other. Mice were placed into one of the arms of the maze and arm entries and time immobile were recorded in real-time for 15 minutes as the animal freely explored all three arms. **Porsolt forced swim** is conducted in a beaker of 23–23 °C water. The water depth ensures that mice can neither touch the bottom not climb out the top. Mice are placed gently into the water and swimming behavior is recorded for 6 minutes. This is repeated 24 hr later to assess memory for the coping strategy (giving up swimming in favor of floating). **Contextual fear conditioning** was conducted to assess fear-based learning and memory. Mice were trained in a chamber (25 × 25 cm) with wire grid flooring inside a sound attenuation chamber with white noise. Mice explored for 3 minutes before being presented with the conditioned stimulus (CS- 90 dB tone) for 30 seconds. The tone co-terminated with the unconditioned stimulus (US- 2 s, 0.57 mA foot shock). Two CS-US pairings were given 90 seconds apart. Mice were placed in the conditioning chamber for 3 minutes at 24 hours after training to assess context learning, and placed in a novel context one hour later. The first 3 minutes in the novel context (pre-CS) measured learning-independent anxiety. Then the CS was presented for 3 minutes in the novel context to test cued learning. All sessions were recorded. Freezing was defined as the absence of movement for 2 consecutive seconds and scored by hand.

### Tissue collection for western blot

To examine CK1δ protein expression in the APP-PS1 brain, a subset of nontransgenic and APP-PS1 mice were treated with vehicle or PF-670462 for 4 days, then euthanized by decapitation. Brains were dissected immediately by scalpel to collect tissue from the prefrontal cortex, anterior hypothalamus, and dorsal hippocampus.

### Western blot quantification of CK1δ

Western blotting was performed as published previously^40^. Brain tissue was lysed in Mammalian Protein Extraction Reagent (M-PER) with phosphatase and protease inhibitors (10 μl/ml, Halt™ Protease and Phosphatase Inhibitor Cocktail, Thermo Scientific Inc, Rockford, IL, USA) using sonication, left to incubate on ice for 15 min. and centrifuged at 20,000 g at 4 °C for 20 min. The supernatant was used for further analysis. Protein concentrations were determined using a BCA assay kit (Thermo scientific). Equal amounts of protein from brain lysate (30ug) were resolved using 9% Tris-glycine polyacrylamide gels under reducing conditions. Proteins were transferred to nitrocellulose membranes (Bio-rad, Hercules CA), and blocked for 1 hour at room temperature in blocking buffer which was a 5% non-fat milk (Labscientific, inc, Livingston, NJ) and tris buffered saline containing 0.1% tween-20 solution (Boston Bioproducts, Ashland, MA). Membranes were then incubated overnight at 4 °C with a polyclonal antibody against CK1δ (Invitrogen), washed 3 × 10 minutes in tris buffered saline with 0.1% tween20 (TBS-T) then placed in blocking buffer combined with HRP-conjugated secondary antibody diluted 1:7500 and left to incubate at room temperature for 1 hour. After thorough washing with TBS-T, bands were visualized using enhanced chemiluminescence (ECL; Thermo scientific) with an image analyzer (Amersham imager 600). Densitometry was performed using Image Lab (Bio-Rad Laboratories, Hercules, CA). Bands of the protein of interest were normalized to an endogenous protein loading control, *beta-actin*.

### Tissue collection for immunofluorescence

To examine amyloid and PER1 expression in the inferior portion of the anterior hypothalamus (the location of the SCN), prefrontal cortex, and hippocampus, the last two cohorts of transgenic mice and age-matched non-transgenic littermates from the circadian and behavioral studies were euthanized at the ending of the experiments, following 14 days of treatment with vehicle or PF-670462. Euthanasia was performed with a solution containing pentobarbital and phenytoin, and mice were transcardially perfused with 25 ml of 0.9% normal saline solution. Mice were placed on an isothermal pad during perfusion to minimize generation of artifactual protein phosphorylation, which has been reported to result from lowered body temperature caused by the euthanizing solution Brains were collected immediately following perfusion and fixed in 4% phosphate-buffered paraformaldehyde for 24 h and then cryoprotected in successive incubations of 10%, 20% and 30% sucrose solutions for 24 h each. Next, brains were frozen and sectioned on a sliding microtome in the coronal plane (30 μm thickness) and stored at 4◦C in Dulbecco’s phosphate buffered saline (PBS) with 10 mM sodium azide solution.

Sections were washed with PBS and blocked with 7% normal horse serum (NHS) in 1.5% Triton-X in PBS for 2 hours. Primary antibody in 7% NHS was applied overnight at 4 °C (anti-beta Amyloid SC-28365 mouse monoclonal antibody, 1:500, Santa Cruz Biotech), or for 48 hours (anti-PER1 PA1–524 rabbit polyclonal antibody 1:250, Invitrogen). The Aβ antibody reacts with amino acids 672–714 of the human Aβ peptide, but due to homology between mouse and human Aβ, there is reactivity in mice as well. Sections were washed, and secondary antibody applied for 2 hours at room temperature (goat anti-rabbit igG (H + L), Alexa Fluor 488, 1:1000, Invitrogen), protected from light. After a final series of washes, sections were mounted on charged slides and allowed to dry for 24 hours before coverslipping (ProLong Gold Antifade Mountant with DAPI nuclear stain, Invitrogen). Sections were imaged at 488 nm and 402 nm using a Zeiss Axioscan.Z1 fluorescent slidescanner Zen 2 software (Zeiss, Thornwood, NY) and quantified in ImageJ (U. S. National Institutes of Health, Bethesda, Maryland). For beta amyloid quantification, plaques were counted in the unilateral hippocampus, defined as well circumscribed fluorescent aggregations with brightness greater than 1 standard deviation higher than mean image brightness. For each subject, the size of the 3 largest plaques was measured in a converted 8-bit image by adjusting threshold to include the area of plaque fluorescence and calculating actual dimensions from the percent area. For PER1, percent expression was measured in cropped, 8-bit images of the unilateral hippocampus or SCN. Threshold was set within 1 SD of the mean brightness, and percent area was measured.

### Data analysis

Data are represented by mean ± SEM. All group sizes were first estimated using g*power *a priori* analysis. For statistical analysis, SPSS version 25 was used. Outliers were first determined – any data points falling outside 2 standard deviations from the group mean were excluded. Next, using the descriptive statistics function, normality was confirmed using the Shapiro-Wilk test for normality (p > 0.05). Due to small sample sizes, data from male and female mice was combined for all analyses. For circadian experiments, data was analyzed by either t-test for baseline differences between APP-PS1 mice and nontransgenic controls or two-way (genotype by drug treatment) repeated measures ANOVA, using 2 time periods: baseline and drug treatment. For behavioral, PER1 IHC, and Western blot experiments, there were only sufficient nontransgenic mouse numbers for a vehicle-treated group, and so one-way ANOVA was used. All significant ANOVA results were further explored by Tukey post-hoc tests. Homogeneity of variance was confirmed using Levene’s test or Equality of Variances in all data sets. Significance was assigned for all tests at p < 0.05.

## Supplementary information

Supplementary Info File #6
