# Author Correction: Inhibition of casein kinase 1δ/εimproves cognitive-affective behavior and reduces amyloid load in the APP-PS1 mouse model of Alzheimer’s disease

**DOI:** 10.1038/s41598-019-51830-5

**Published:** 2019-10-17

**Authors:** S. Sundaram, S. Nagaraj, H. Mahoney, A. Portugues, W. Li, K. Millsaps, J. Faulkner, A. Yunus, C. Burns, C. Bloom, M. Said, L. Pinto, S. Azam, M. Flores, A. Henriksen, J. Gamsby, D. Gulick

**Affiliations:** Department of Molecular Medicine and Byrd Institute, University of South Florida, Tampa, FL USA

Correction to: *Scientific Reports* 10.1038/s41598-019-50197-x, published online 24 September 2019

The Acknowledgements section in this Article is incomplete.

“This work was made possible by a grant from the Florida Department of Health (7AZ13; DG) and by stipends from the Morsani College of Medicine Research, Innovation & Scholarly Endeavors Office (SD; SN).”

should read:

“This work was made possible by grants from the Florida Department of Health (7AZ13, DG; 7AZ24 and 9AZ31, JG), Alzheimer’s Association (2016-NIRG-393975, JG) and by stipends from the Morsani College of Medicine Research, Innovation & Scholarly Endeavors Office (SS; SN).”

